# Acute Effects of Tissue Flossing on Knee Flexion Range of Motion in Healthy Adults: A Randomized Controlled Trial

**DOI:** 10.3390/jcm15124718

**Published:** 2026-06-17

**Authors:** Ricardo Cardoso, Maëva Bosquier, Isabel Moreira-Silva, Joana Azevedo, Adérito Seixas

**Affiliations:** 1 FP-I3ID, FP-BHS, Escola Superior de Saúde Fernando Pessoa, 4200-256 Porto, Portugal; mbosquier@icloud.com (M.B.); isabelmsilva@ufp.edu.pt (I.M.-S.); jsazevedo@ufp.edu.pt (J.A.); aderito@ufp.edu.pt (A.S.); 2 CIAFEL, Faculdade de Desporto, Universidade do Porto, 4200-450 Porto, Portugal; 3 LABIOMEP, INEGI-LAETA, Faculdade de Desporto, Universidade do Porto, 4200-450 Porto, Portugal

**Keywords:** tissue flossing, range of motion, knee joint, blood flow restriction, randomized controlled trial

## Abstract

**Background/Objectives**: Tissue flossing (TF) with elastic bands (floss bands) is a therapeutic strategy to improve joint range of motion (ROM). While TF has demonstrated 3–7% ROM improvements in ankle and shoulder joints, its effects on knee flexion remain underexplored. Therefore, the objective of this study was to investigate the acute effects of TF on active and passive knee flexion range of motion in healthy adults. **Methods**: Sixty healthy participants (median age 23.0 [IQR 2.0] years; 30 male, 30 female) were randomized to an intervention group (IG; *n* = 30) receiving floss band (COMPRE Sanctband^®^, Level 1; 50% tension, 50% overlap) application combined with knee mobilization (20 active/passive repetitions over 2 min), or a control group (CG; *n* = 30) performing the same mobilization without band application. Active (AROM) and passive (PROM) knee flexion were measured pre- (M0) and post-intervention (M1) using a validated smartphone goniometer (Goniometer Pro), by a blinded assessor. **Results**: Baseline characteristics (age, body mass index) did not differ between groups (*p* > 0.05); however, baseline AROM differed significantly between groups (*p* = 0.041). The IG showed significantly greater improvements than CG in AROM (Δ5.0° [4.0%] vs. Δ0.0°, *p* < 0.001) and PROM (Δ6.0° [4.5%] vs. Δ1.0° [0.8%], *p* < 0.001). **Conclusions**: TF combined with mobilization produced greater immediate increases in knee flexion ROM than mobilization alone, with large effect sizes. These findings support adequately powered, sham-controlled trials in clinical populations before clinical effectiveness can be inferred.

## 1. Introduction

Tissue flossing (TF), also known as the use of floss bands (FB), is a novel therapeutic intervention that combines mechanical compression with movement to potentially enhance joint range of motion (ROM), reduce pain, and improve neuromuscular performance. This method involves applying an elastic band around joints or muscle groups with controlled tension and overlap, typically ranging from 50% to 90% elongation [1,2]. The technique derives conceptually from blood flow restriction (BFR) training methods such as Kaatsu, developed in Japan in the 1960s [3], and has gained increasing attention in the fields of sports therapy and rehabilitation [4,5].

TF has been shown to provide various benefits, including acute improvements in ROM, reductions in muscle stiffness, and enhanced functional performance such as jump height or sprint time [6,7]. Most available research has focused on joints such as the ankle [8], shoulder [1], and soft tissues including the calf [9] and thigh [7]. In these contexts, TF has demonstrated acute ROM improvements ranging from approximately 3% to 7%, depending on the joint and protocol employed [1,6,9]. However, evidence regarding the knee joint remains limited. Only a few studies have examined TF applied directly around the knee, reporting potential benefits in muscle flexibility [10,11] and pain reduction [12]. Nevertheless, the effects on knee joint range of motion, particularly in the immediate post-intervention period, remain poorly documented.

This is particularly relevant since knee ROM plays a critical role in fundamental activities of daily living. Functional flexion angles of the knee range from approximately 67° for walking to 105° for tasks such as tying shoelaces [13]. Loss of knee mobility is a common impairment following injury, surgery, or due to musculoskeletal restrictions, leading to reduced function, compensatory movement patterns, and diminished quality of life [14]. The fascial and muscular structures influencing knee motion—such as the quadriceps, hamstrings, gluteus maximus, and iliotibial band—form a complex kinetic chain in which tension balance is essential for both performance and injury prevention [15].

Several mechanisms have been proposed to explain TF-induced ROM gains, including myofascial shearing, tissue thixotropy, reactive hyperemia following band release, gate-control analgesia, and mobilization of fascial layers restricting joint movement [6,16,17]. These mechanisms remain largely theoretical; however, they collectively provide a plausible biological rationale for the technique. Importantly, TF effects may vary by joint due to differences in tissue depth, compression tolerance, vascular anatomy, and fascial architecture, underscoring the need for joint-specific investigation.

Despite these proposed mechanisms, targeted evidence on knee flexion ROM remains scarce. Wu et al. (2022) [11], in a randomized crossover trial, demonstrated that applying TF around the knee significantly improved hamstring flexibility in physically active female participants without compromising balance or hop performance, with effects persisting for up to 20 min post-intervention. Similarly, Chang et al. (2021) [10] reported increases in flexibility, proprioception, and strength following TF application to the thigh area, which is biomechanically linked to knee mobility. In clinical contexts, a randomized controlled trial on boxers with chronic knee pain found that a 3 min tissue flossing application yielded significant acute improvements in pain, function, extensor strength, and joint stability. Although knee flexion ROM showed a mean increase of 6.32°, this improvement did not reach statistical significance [18]. Furthermore, an experimental study by Rodrigo-Mallorca et al. (2024) [19] demonstrated that TF applied to the ankle region could acutely enhance dorsiflexion and jump performance, suggesting a potential systemic or chain-related benefit of the technique even when applied away from the primary joint of interest.

Recent systematic reviews further emphasize the pertinence of investigating TF. Cheatham et al. (2024) [20] synthesized emerging evidence on athletic performance, concluding that TF applications of 2 to 10 min can enhance muscle strength, jump performance, and balance for up to 60 min post-intervention, despite wide variability in protocols. Similarly, Tedeschi and Giorgi (2024) [21] reported statistically significant improvements in mobility and muscle function, particularly at the ankle and hamstring regions. Most recently, a comprehensive meta-analysis by Yao et al. (2026) [22] confirmed a trivial but significant acute increase in joint ROM (Hedges’ g = 0.19) across 20 studies. Crucially, this latest evidence identifies that TF’s efficacy is highly dependent on specific moderators, showing better results when applied to the lower limbs, using low wrapping pressures (≤150 mmHg), and in trained populations. However, despite these advancements, targeted research specifically examining knee ROM remains less prevalent than studies on the ankle, justifying further investigation into this specific joint complex.

Therefore, the aim of the present study was to evaluate the immediate effects of TF applied around the dominant knee joint on active and passive knee flexion ROM in healthy adults.

## 2. Materials and Methods

This report adhered to the CONSORT guidelines for reporting clinical trials.

### 2.1. Ethical Considerations

The protocol was approved by Fernando Pessoa University’s Ethics Committee (ESS/FSA-320/22) and registered in ClinicalTrials.gov (NCT06205069). All participants provided written informed consent following the ethical principles outlined in the Declaration of Helsinki. Participants were informed about the study’s purpose, procedures, and their right to withdraw at any time. Confidentiality and anonymity were strictly ensured. Data collection took place between 15 February 2024 and 12 March 2024.

Prior to the start of recruitment, the intervention protocol (50% tension and 50% overlap) and the mobilization exercises were pilot-tested with a small group of healthy volunteers (*n* = 3) to ensure technical feasibility and application comfort. No modifications were made to the original trial design following this consultation.

### 2.2. Participants

Sixty healthy volunteers (30 male, 30 female), aged 18 to 35 years, were recruited for this study. The choice of a healthy participant sample was deliberate: it allows the biological effect of TF to be isolated from confounding factors such as inflammation, post-surgical fibrosis, or pain-related inhibition of movement, and mirrors the proof-of-concept approach used in prior TF studies at the ankle and shoulder. Participants of both sexes were included if they demonstrated normal joint mobility in the lower body quadrant [9], as assessed through active movements of the spine, hips, knees and feet. Normal joint mobility was defined according to the reference values established by Norkin and White (2016) [23], which include, for the knee, an active flexion range of 135° and full extension to 0°. Each movement was visually assessed by the examiner to ensure it fell within these normative values and was free from compensatory patterns. Exclusion criteria comprised: deformities in the region of the lower limbs of the body; complaints in this region in the last six months; surgical procedures; venous thrombotic disease; heart disease; respiratory disease; or neurological, orthopedic, dermatitis, or neuromuscular problems in the lower quadrant that may disrupt musculoskeletal function. Also, individuals with high blood pressure, latex allergies, lymphedema and taking anticoagulant medication, or corticosteroid therapy were excluded [11].

### 2.3. Randomization

Participants were randomly divided into two groups using the GraphPad online tool (https://www.graphpad.com/quickcalcs/, accessed on 15 February 2024). A simple randomization method was used, with no blocking or stratification. The random allocation sequence was generated by one investigator prior to recruitment. Allocation concealment was ensured through the use of sequentially numbered, sealed, opaque envelopes, opened only after each participant’s baseline assessment was completed. The same investigator who generated the sequence also enrolled participants and assigned them to groups. Participants were allocated to an Intervention Group (IG, *n* = 30), receiving TF with mobilization, or a Control Group (CG; *n* = 30), performing mobilization alone.

### 2.4. Protocol

All participants completed a Characterization Sociodemographic and Clinical Questionnaire to identify possible exclusion criteria, which included personal data (2 items), training habits (1 item) and clinical background (9 items). Anthropometric measurements, including height and weight, were recorded to calculate body mass index (BMI). The procedures were carried out on the dominant lower limb. To identify the dominant lower limb, a test was carried out in which a ball (Kipsta^®^, Decathlon, Villeneuve-d’Ascq, France) was thrown to the participant, who had to return it using the lower limb [24].

Measurements were collected at baseline (M0) and immediately after the intervention (M1). Knee flexion range of motion (ROM) was assessed for all participants using a smartphone goniometer application (iPhone 11, iOS 16, Apple Inc., Cupertino, CA, USA), following established methodology [25]. The measurement protocol required participants to assume a prone position with the hip and knee of the dominant limb extended to 0°, the foot relaxed, and the contralateral leg extended, thus defining the goniometer’s zero starting position [23].

The Goniometer Pro application (v2.8) was used, leveraging the smartphone’s embedded inertial sensors (accelerometer and gyroscope) to calculate joint angles based on the device’s precise position and orientation in space. This hardware–software system is a validated tool, demonstrating high reliability and accuracy for determining knee flexion angles, with studies indicating slightly greater precision than traditional goniometry [26,27]. Its use also yields superior intra- and inter-observer correlation scores [28]. A systematic review of 37 studies further confirms that smartphone applications are reliable and valid instruments for measuring joint ROM in clinical settings [29].

For consistent placement, the long edge of the smartphone was aligned along the anatomical axis connecting the lateral femoral epicondyle and the lateral malleolus. The distance between these two landmarks was recorded at the initial position (M0) to ensure identical device placement for subsequent measurements on the same knee, thereby safeguarding intra-observer reliability.

**Figure 1:**
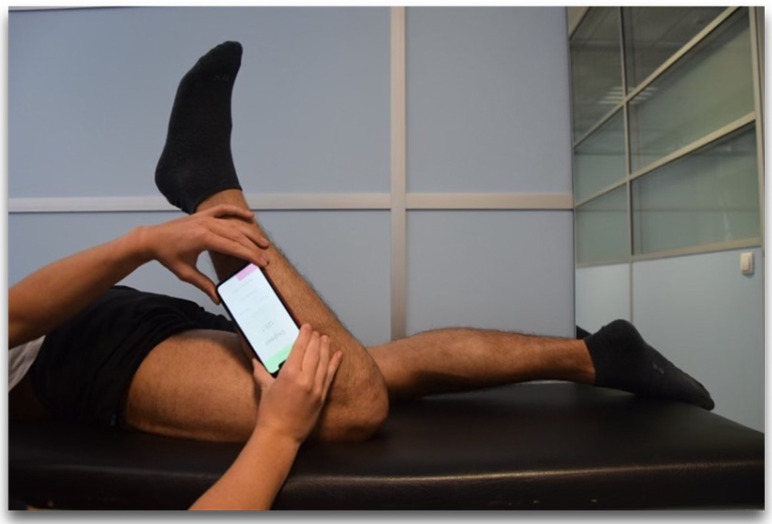
Measurement of knee flexion ROM using smartphone goniometry.

The application’s operation involves tapping the touchscreen to set an initial angle (0°); following movement of the limb, a second tap at the terminal position records the angular difference [25]. Both active and passive knee flexion were measured. For active ROM, participants were instructed to perform maximal knee flexion while maintaining the prone position. For passive ROM, the examiner performed maximal knee flexion for the participant. Each measurement type (active and passive) was repeated three times with a 30 s rest interval between trials to prevent muscle fatigue (Figure 1). The mean value of the three trials was used as the final ROM score for each measurement type and time point, in accordance with established protocols for goniometric assessments [25].

To ensure data consistency and internal validity, all ROM measurements at both baseline (M0) and post-intervention (M1) were performed by the same trained examiner for each participant. Regarding blinding, although it was not possible to blind the participants due to the physical nature of the elastic band application, the assessor responsible for recording the angles via the Goniometer Pro application remained blinded to the group allocation throughout the entire procedure.

### 2.5. Intervention

Following the initial assessment (M0), a floss band (FB) was applied to the knee of the dominant lower limb in the intervention group (IG). Participants assumed a semi-seated position with the dominant leg in slight forward flexion [9].

**Figure 2:**
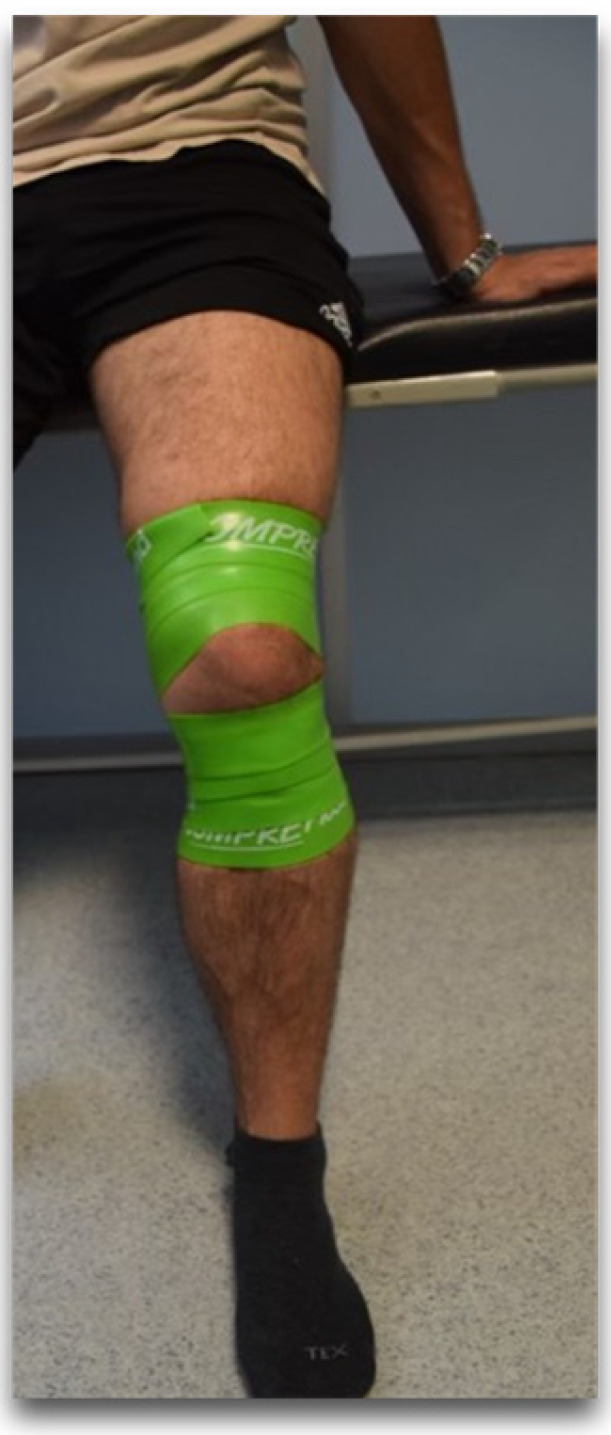
Floss band application.

The FB used in this study was a natural rubber COMPRE Floss band (Sanctband^®^, Ipoh, Malaysia). The specific band was Level 1 (green), 5 cm in width (5 cm × 206 cm; Sanct Japan Co., Ltd., Tokyo, Japan). The wrapping technique followed the manufacturer’s guidelines (Sanctband^®^ user manual). While the participant stood with slight knee flexion, the band was applied proximally, starting from the tibial tuberosity as the distal anchor point and wrapping up to approximately 5 cm above the femoral epicondyle. The patella was left uncovered. Pressure was applied by wrapping the joint with 50% tension and a 50% overlap between successive turns [6], a method designed to apply pressure to both the joint and surrounding soft tissues (Figure 2).

Immediately after application, participants assumed a supine position and performed a standardized movement protocol. This consisted of:

1. A passive movement task: 20 repetitions of passive knee flexion and extension performed by the examiner.
2. An active movement task: 20 repetitions of active knee flexion and extension through their full range of motion, followed by bodyweight squats [4].

Participants were instructed to complete all mobility exercises within a two-minute timeframe [8]. After two minutes, the FB was removed. Participants were then instructed to stand and walk for one minute to facilitate the return of blood flow to the foot [8].

For the control group (CG), following the initial assessment, participants performed the identical two functional movement tasks (active and passive with 20 repetitions each) for two minutes without the application of the FB. Subsequently, they also stood and walked for one minute, mirroring the protocol of the intervention group.

Potential harms or adverse events (e.g., persistent paresthesia, excessive pain, or skin alterations) were systematically assessed through direct observation during the 2 min FB application and via immediate verbal questioning after band removal and the subsequent one-minute walk.

A post-intervention assessment (M1) was performed immediately after the intervention or control procedures for all participants. All assessments (M0 and M1) for a given participant were conducted by the same examiner to ensure consistency.

### 2.6. Statistical Procedures

Data analysis was performed using the Statistical Package for the Social Sciences (IBM SPSS Statistics, Version 29.0) for macOS. The normality of the data distribution for continuous variables was assessed using the Kolmogorov–Smirnov test, which indicated a significant deviation from normality. Consequently, non-parametric tests were employed for all continuous variable analyses.

Continuous variables (age and body mass index) are presented as median (Me) and interquartile range (IQR), and inter-group comparisons were performed using the Mann–Whitney U test. Nominal variables (sex and lower limb dominance) are presented as absolute frequency and percentage [*n* (%)], and inter-group comparisons were performed using the Chi-square (χ^2^) or Fisher’s exact test. For intra-group comparisons of ROM outcomes between pre-intervention (M0) and post-intervention (M1) measurements, the Wilcoxon signed-rank test was applied. The rank biserial correlation (rrb) was used as a measure of ES in Mann–Whitney and Wilcoxon tests, and absolute values were considered small (0.1 < ES < 0.3), medium (0.3 ≤ ES < 0.5), or large (0.5 ≤ ES < 1). A *p*-value of <0.05 was considered statistically significant for all tests. No participants were lost to follow-up; all 60 randomized participants completed the study and were included in the analysis (complete-case analysis). No subgroup or sensitivity analyses were performed.

## 3. Results

### Sample Characteristics

**Figure 3:**
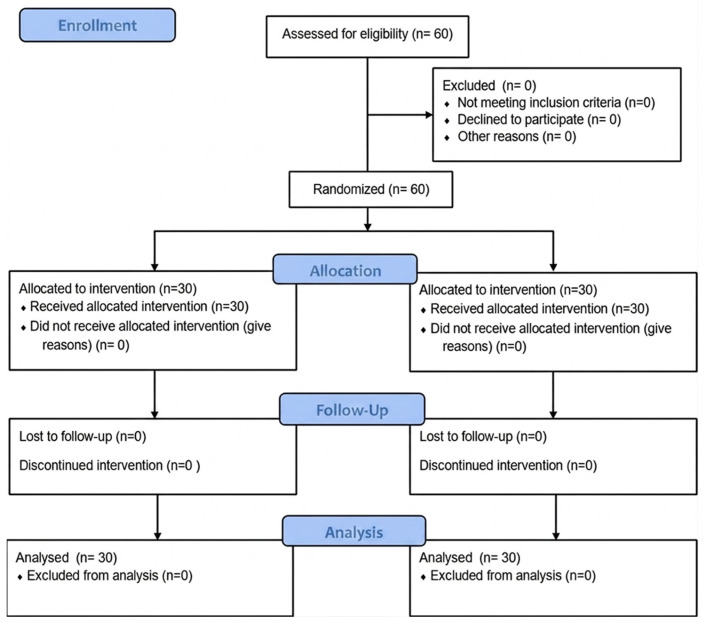
CONSORT flow diagram.

A total of 60 participants were included in this study, with a median age of 23.0 (IQR: 2.0) years and a median BMI of 22.80 (IQR: 3.97) kg/m^2^. Both the control group (CG) and the intervention group (IG) consisted of 30 healthy participants each. The CG and IG each comprised 15 males (50%) and 15 females (50%). Regarding lower limb dominance, 28 participants (93.3%) in both the CG and IG were right-dominant. No statistically significant differences were found between groups for sociodemographic or anthropometric characteristics (age, BMI, sex, and lower limb dominance; *p* > 0.05; Table 1). A CONSORT flow diagram is presented in Figure 3. No adverse events were reported during data collection. All 60 enrolled participants were randomly allocated (30 per group) and all completed the study protocol; there were no withdrawals, dropouts, or exclusions after randomization.

A flow diagram of this parallel randomized controlled trial is presented in Figure 3.

Analysis of knee flexion range of motion revealed a statistically significant difference between groups in AROM at baseline (M0; IG: 126.0° [IQR: 9.5] vs. CG: 122.0° [IQR: 8.8]; *p* = 0.041, rᴦ = −0.308), with the IG presenting higher values. No significant baseline difference was found for PROM (*p* = 0.177; Table 2).

Following the intervention, a significant within-group improvement was observed for AROM in both the IG (*p* < 0.001, rᴦ = −1.000) and the CG (*p* = 0.003, rᴦ = −0.794). Similarly, a significant within-group improvement in PROM was found for both the IG (*p* < 0.001, rᴦ = −1.000) and the CG (*p* = 0.004, rᴦ = −0.654).

However, when comparing the groups at the post-intervention assessment (M1), the IG demonstrated a significantly greater improvement in both active and passive knee flexion ROM compared to the CG (*p* < 0.001). The median change from baseline (M1−M0) was 5.0° (IQR: 0.8) for AROM and 6.0° (IQR: 2.0) for PROM in the IG, compared to 0.0° (IQR: 1.0) for AROM and 1.0° (IQR: 1.0) for PROM in the CG. The rank biserial correlations for the inter-group difference in change scores were rᴦ = −0.997 for AROM and rᴦ = −1.000 for PROM, indicating a large effect size.

## 4. Discussion

### 4.1. Findings of the Present Study

The present study demonstrated that a single application of TF combined with standardized mobilization exercises resulted in significantly greater improvements in both active and passive knee flexion ROM compared to mobilization alone. Although both groups exhibited within-group improvements, the magnitude of change was substantially greater in the intervention group, suggesting an additive effect of TF beyond movement-based interventions. It should be noted, however, that participants were healthy young adults with near-normal baseline ROM; in this context, the observed acute change should be interpreted with caution, as it may partly reflect transient warm-up, stretching, or familiarization effects rather than solely a unique therapeutic action of TF, and may not directly extrapolate to clinical populations with genuine ROM deficits.

A noteworthy finding was the statistically significant within-group improvement observed in the CG for both AROM and PROM, despite the absence of FB application. This result warrants careful interpretation and may reflect several non-specific mechanisms: a motor learning or familiarization effect arising from repeated performance of the same movement pattern across assessments; an acute warm-up effect resulting from two minutes of active and passive knee mobilization, which could transiently reduce tissue viscosity and increase joint lubrication; or a genuine exercise-induced improvement in ROM driven by the movement protocol itself. These possibilities are not mutually exclusive. This finding is consistent with observations from Kiefer et al. (2017) [1], Vogrin, Kalc & Ličen (2020) [7], and Mills et al. (2020) [30], who also re-ported ROM gains in control groups performing movement without FB. Importantly, the magnitude of change in the CG was substantially smaller than in the IG (AROM: Δ0.0° vs. Δ5.0°; PROM: Δ1.0° vs. Δ6.0°), and the inter-group difference was statistically significant, indicating that the compression component of TF confers a meaningful additive benefit beyond exercise alone. Nonetheless, future studies employing passive rest control conditions, rather than active exercise controls, would help to disentangle these effects more precisely.

The observed increase of approximately 5–6° in knee flexion may indicate a clinically meaningful acute effect. Although established Minimal Clinically Important Difference (MCID) values for knee flexion ROM have been primarily derived from post-surgical populations, where thresholds of 5–10° are commonly reported [14], their direct applicability to healthy adults remains uncertain. In the absence of population-specific MCID benchmarks for this context, the 5–6° change observed in the IG should be interpreted conservatively. The finding that the improvement meets or exceeds the lower bound of the post-surgical MCID range suggests that it may be clinically relevant. However, confirming its clinical meaningfulness would require longitudinal data, patient-reported outcome measures, and MCID thresholds specifically established for healthy adults, which are not yet available for this context.

A baseline difference in AROM was observed between groups (IG: 126.0° vs. CG: 122.0°; *p* = 0.041), with the IG presenting higher values despite randomization. Although this difference was statistically significant, its clinical magnitude is modest (approximately 4°). Additionally, considering that the MDC for smartphone goniometry of knee flexion has been reported as 4° [31]. Nonetheless, the possibility that ceiling effects or regression-to-the-mean may have differentially influenced the IG—which started with higher values—cannot be fully excluded. The primary inter-group analysis relied on change scores (M1−M0) rather than absolute post-intervention values, which partially mitigates this confound.

The rank biserial correlation (rᴦ), used as the effect size measure for both intra- and inter-group comparisons, provides an important complement to *p*-values by quantifying the practical magnitude of the observed differences. Within-group, the IG showed a large effect for both AROM and PROM (rᴦ = −1.000), indicating that all participants improved from M0 to M1 after tissue flossing with mobilization. The CG also showed large within-group effect sizes for AROM (rᴦ = −0.794) and PROM (rᴦ = −0.654), consistent with the known warm-up and mobilization effects discussed above. Crucially, the inter-group comparison of change scores revealed a large effect for both AROM (rᴦ = −0.997) and PROM (rᴦ = −1.000), confirming that the superiority of the TF intervention was not only statistically significant but also of substantial practical magnitude. These effect sizes strengthen confidence in the additive benefit of the compression component beyond exercise alone and support the clinical relevance of the intervention within the constraints of this trial. We acknowledge that rank-biserial correlations of this magnitude (rᴦ = −1.000) are un-common in biological research and warrant cautious interpretation. Such values arise when there is complete separation between groups in the ranked change scores (i.e., every IG participant exhibited a larger improvement than every CG participant), which can occur in small, homogeneous samples with a constrained range of possible ordinal outcomes (changes recorded in whole-degree increments). This pattern may reflect a genuine and consistent effect of TF in this sample, but could also be influenced by the limited sample size, measurement granularity, and absence of a sham condition. These effect sizes should therefore be regarded as hypothesis-generating rather than definitive, and replication in larger, independent samples with bootstrap confidence intervals is recommended.

### 4.2. Comparison with Previous Studies

Several studies have explored the acute effects of FB on joint ROM. Driller and Overmayer (2017) [6] reported a 7.37% increase in ankle dorsiflexion after 2 min of active ankle movements with FB, while Kaneda et al. (2020) [9] observed a 32.9% increase in dorsiflexion after FB application to the calf. Most research has focused on joints such as the ankle or shoulder [1], with fewer studies addressing the knee.

Among studies involving the knee, Chang et al. (2021) [10], reported improvements in hamstring (5.4%) and quadriceps (2.24%) flexibility following FB application to the thigh, assessed through flexibility tests rather than direct goniometric measurement of joint angle. Wu et al. (2022) [11] found a 3.74% increase in active knee ROM after applying FB around the knee in healthy female participants, measured using a standardized knee flexion test; however, this was expressed as a percentage change in a functional performance score rather than as an absolute goniometric joint angle, which limits direct comparison with the present findings. The current study extends this evidence by providing both active and passive knee ROM values in degrees, measured with a validated smartphone goniometer applied directly to bony landmarks, thereby offering a more precise and clinically interpretable quantification of the effect.

Additionally, studies by Kiefer et al. (2017) [1], Vogrin, Kalc & Ličen (2020) [7], and Mills et al. (2020) [30] reported similar ROM improvements in control groups that performed stretching or movement without FB, suggesting that movement alone may have conditioning effects. However, the greater magnitude of improvement observed in our FB group indicates a potential additive benefit of compression.

Regarding clinical applications of TF to the knee, a recent randomized controlled trial integrating FB into conventional physiotherapy for patellofemoral pain syndrome demonstrated superior outcomes in pain reduction, muscle strength, and lower extremity function compared to standard physiotherapy alone [32]. Additionally, Chen et al. (2025) [18] investigated the acute effects of TF on boxers with chronic knee pain, reporting significant improvements in pain (VAS), knee function (Lysholm score), and maximal isometric extensor strength. Regarding knee flexion ROM specifically, no statistically significant inter-group difference was found, suggesting that TF’s acute effect on ROM may be more pronounced in healthy populations without pre-existing joint pathology, or may require different protocol parameters in clinical contexts. This further underscores the clinical relevance of knee-focused TF research in both healthy and clinical populations.

Recent systematic reviews highlight the emerging but still fragmented evidence regarding TF. While Cheatham et al. (2024) [20] and Tedeschi and Giorgi (2024) [21] acknowledge improvements in lower limb flexibility and muscle function, they emphasize significant methodological heterogeneity and a relative scarcity of high-quality studies focused specifically on knee joint ROM compared to the ankle. This landscape was further clarified by the most recent meta-analysis from Yao et al. (2026) [22], which reported a trivial but significant acute increase in joint ROM (Hedges’ g = 0.19). Crucially, Yao et al. (2026) [22] identified that TF efficacy is highly dependent on specific moderators, showing superior results when applied to the lower limbs and using low wrapping pressures (≤150 mmHg). The continued lack of standardized protocols specifically targeting knee flexion ROM underscores the relevance of the present investigation, which addresses this literature gap by providing a controlled assessment of immediate effects using a standardized application method.

### 4.3. Possible Mechanisms

Several physiological mechanisms may explain the ROM gains observed. First, the thixotropic effect may reduce tissue viscosity via fluid redistribution and collagen fiber reorganization, allowing greater fascial and muscular glide [4]. Second, FB may enhance stretch tolerance and reduce pain through gate control mechanisms [16]. Third, FB may stimulate proprioceptive and interstitial receptors, promoting reflex muscle relaxation and improved tissue compliance. Lastly, it may help to mobilize fascial layers and break down adhesions that restrict joint mobility [33]. A recent conceptual framework has proposed the terms “Pain Gate,” “Fascia Glide,” “Sponge Effect,” “Soft Tissue Ischemia,” and “Joint Effect” as descriptive labels for these putative mechanisms. It should be emphasized that this framework is largely narrative and has not been validated through direct physiological measurement; it is presented here as a conceptual organizing structure rather than as established mechanistic evidence [17].

Collectively, these effects support the use of FB as a strategy to acutely increase ROM. In clinical contexts, quadriceps flexibility is crucial for posture, movement efficiency, and injury prevention. Previous research has associated limited quadriceps flexibility with patellar tendinopathy [34] and low back pain in adolescents [35]. Enhanced quadriceps flexibility may therefore benefit both rehabilitation and athletic performance, particularly in populations with knee mobility restrictions or musculoskeletal pathologies [36].

Importantly, none of the mechanisms discussed above (myofascial shearing, thixotropy, hyperemia, gate-control analgesia, or fascial mobilization) were directly measured in the present study, and the exact mechanisms underlying TF-induced ROM changes therefore remain speculative. Current evidence for these mechanisms is largely theoretical or extrapolated from studies of related manual and compression techniques. Future studies incorporating direct physiological measurements (e.g., blood flow via Doppler ultrasound, tissue stiffness via myotonometry or elastography, electromyography) are required to test these hypotheses empirically before they can be considered explanatory rather than illustrative.

### 4.4. Clinical Implications

From a practical perspective, TF represents a low-cost, time-efficient, and easily applicable intervention that may be used as an adjunct to traditional mobility or rehabilitation programs. The acute improvements observed in this study suggest potential utility in warm-up routines, injury prevention strategies, or preparatory phases of rehabilitation. Available evidence from related TF studies suggests the ROM gains may persist long enough to benefit a subsequent session: Wu et al. (2022) [11] reported effects lasting up to 20 min, and Cheatham et al. (2024) [20] documented performance benefits for up to 60 min post-intervention. Since the present study included no follow-up assessment, the temporal stability of the observed effects remains unknown. Future studies should incorporate delayed time-point assessments (e.g., 15 and 30 min post-intervention) to define the practical window of effect.

However, given the short-term nature of the effects and the limited evidence in clinical populations, TF should not be considered a standalone intervention. Its application should be integrated within a broader, evidence-based rehabilitation framework. To address the translational applicability of these findings more concretely, we pro-pose the following clarifications. Regarding target populations: based on the association between quadriceps flexibility and conditions such as patellar tendinopathy and adolescent low back pain discussed above, individuals presenting with reduced quadriceps/anterior thigh flexibility and consequent restrictions in knee flexion ROM—rather than the general healthy population studied here—represent the most plausible target group. This may include athletes during return-to-sport phases, individuals recovering from prolonged immobilization, or patients with documented quadriceps strength and flexibility deficits, such as those described following patellar dislocation (Biz et al., 2024) [37]. Regarding the rehabilitation phase: based on the acute, short-duration nature of the observed effect, TF appears best suited as a preparatory adjunct immediately preceding therapeutic exercise, stretching, or functional training (i.e., within a warm-up or pre-mobilization window), rather than as a stand-alone or maintenance therapy. Regarding comparison with conventional techniques: in the present study, TF combined with movement produced significantly greater ROM gains than movement alone, suggesting a potential incremental benefit over conventional stretching or mobilization; however, head-to-head trials directly comparing TF with standardized stretching protocols, using equivalent time and con-tact exposure, are needed before comparative claims can be made. Regarding safety and contraindications: consistent with guidance for other compression-based techniques (e.g., blood flow restriction training), TF should be avoided or used with extreme caution in individuals with a history of deep vein thrombosis or other clotting disorders, peripheral vascular disease, varicose veins, lymphedema, compromised skin integrity, sensory impairment, uncontrolled hypertension, or acute injury/inflammation in the targeted limb. Pressure and application duration should be conservative until joint- and population-specific safety data are available.

### 4.5. Limitations

This study presents several limitations. First, the intervention was short-term with no follow-up; the durability of the observed ROM gains beyond the immediate post-intervention period remains unknown. Second, and critically, compression pressure was not objectively quantified with a Doppler device or pressure cuff. In-ter-individual variation in band tension is an inherent limitation of current TF protocols and represents a major barrier to reproducibility. Yao et al. (2026) [22] identified wrapping pressure (≤150 mmHg) as a key moderator of TF efficacy; future studies must incorporate pressure monitoring to enhance external validity. Third, the sample was restricted to healthy young adults, limiting generalizability to clinical populations. Fourth, a statistically significant baseline AROM imbalance was observed (IG: 126.0° vs. CG: 122.0°; *p* = 0.041); while change scores were used for the primary analysis, ceiling effects or regression-to-the-mean cannot be excluded. Fifth, the sample size was based on prior comparable studies rather than a formal a priori power calculation; the absence of this calculation limits our ability to characterize statistical precision. The observed large effect sizes provide an empirical basis for powering future trials. Future studies should incorporate pressure calibration, stratified randomization, a priori power analyses, adverse-event monitoring, delayed follow-up assessments, and enrolment of clinical populations. Sixth, the trial did not include a sham or placebo-band condition: the CG performed the movement protocol without any band, rather than with a sham wrap applied at minimal, non-therapeutic tension. Consequently, non-specific effects related to tactile stimulation, skin pressure, or participant expectation cannot be disentangled from the true mechanical/compressive effect of TF. This represents an important methodological constraint for a manual, compression-based intervention, and future trials should include a minimal-tension sham-wrap arm to address this issue.

### 4.6. Future Directions

Future research should aim to standardize TF application parameters, including pressure, duration, and movement protocols. Randomized controlled trials with larger and more diverse samples, including clinical populations, are needed to determine the effectiveness of TF in rehabilitation settings. In particular, future trials should be formally powered based on the large effect sizes observed in the present study (rr_b = −0.997 for AROM; rr_b = −1.000 for PROM), and should incorporate objective pressure monitoring, sham-controlled designs, and patient-reported outcomes to improve methodological rigor.

Longitudinal studies are particularly important to assess the duration of effects and potential cumulative benefits of repeated applications. Additionally, studies integrating biomechanical and physiological outcomes may help clarify the mechanisms underlying TF and optimize its clinical use.

## 5. Conclusions

Within the limitations of a small, short-term randomized trial in healthy young adults with near-normal baseline ROM, tissue flossing combined with a standardized movement protocol produced significantly greater immediate increases in active and passive knee flexion ROM than the movement protocol alone. Given the absence of a sham-band condition, the healthy and high-functioning nature of the sample, and the lack of follow-up assessment, these findings should be regarded as preliminary, proof-of-concept evidence rather than support for specific clinical indications. They do not, on their own, establish that TF confers a clinically meaningful advantage over conventional movement-based or stretching interventions in clinical populations. Adequately powered, sham-controlled trials incorporating objective pressure calibration, longer follow-up, adverse-event reporting, and enrolment of clinical populations with genuine ROM deficits are required before specific clinical recommendations regarding the indications, target populations, and rehabilitation phase for TF can be formulated.

## Figures and Tables

**Table 1:** Characteristics of the participants of both groups.

Variables	Control Group (CG) (*n* = 30)	Intervention Group (IG) (*n* = 30)	*p*
Age (years) ^a^	23.0 (2.0)	23.5 (2.8)	0.508
Body mass index (kg/m^2^) ^a^	22.9 (4.0)	22.2 (3.5)	0.404
Sex ^b^			
Male	15 (50.0%)	15 (50.0%)	1.000
Female	15 (50.0%)	15 (50.0%)	
Dominant lower limb ^c^			
Right	28 (93.3%)	28 (93.3%)	1.000
Left	2 (6.7%)	2 (6.7%)	

^a^ Presented as median (interquartile range)—Me (IQR); compared using the Mann–Whitney U test. ^b^ Presented as *n* (%); compared using the Chi-square test (χ^2^). ^c^ Presented as *n* (%); compared using the Fisher exact test (χ^2^).

**Table 2:** Intra- and inter-group differences in knee flexion range of motion before (M0) and after the intervention (M1).

Variables	Group	M0 (AROM)	M1 (AROM)	*p* #	r_rb_	M0 (PROM)	M1 (PROM)	*p* #	r_rb_
		Me; IQR; Min–Max	Me; IQR; Min–Max			Me; IQR; Min–Max	Me; IQR; Min–Max		
Knee flexion (°)	CG	122.0; 8.8; 115–140	122.0; 7.8; 114–141	0.003 *	−0.794	128.0; 9.8; 120–150	129.0; 11.5; 120–151	0.004 *	−0.654
	IG	126.0; 9.5; 118–136	131.0; 10.0; 122–140	<0.001 *	−1.000	134.0; 11.5; 122–150	140.0; 11.8; 125–155	<0.001 *	−1.000
	*p* ^†^	0.041 *	<0.001 *			0.177	<0.001 *		
	r_rb_	−0.308	−0.658			−0.203	−0.532		
Difference in Knee Flexion (°) (M1–M0).	CG	0.0; 1.0; −1–2				1.0; 1.0; −1–2			
	IG	5.0; 0.8; 2–11				6.0; 2.0; 3–10			
	*p* ^†^	<0.001 *				<0.001 *			
	r_rb_	−0.997				−1.000			

* Significant values (*p* ≤ 0.05); *p* # for significant intra-group differences—Wilcoxon test; *p* ^†^ for significant inter-group differences—Mann–Whitney test. Abbreviations: CG: Control Group; IG: Intervention Group; AROM: Active Range of Motion; PROM: Passive Range of Motion; r_rb_: The rank biserial correlation.

## Data Availability

De-identified individual participant data, the data dictionary, and the statistical code used for analyses in SPSS (v. 29.0) will be made available upon reasonable request to the corresponding author following publication, for a period of five years.
